# Views of advanced cancer patients, families, and oncologists on initiating and engaging in advance care planning: a qualitative study

**DOI:** 10.1186/s12904-020-00655-5

**Published:** 2020-10-01

**Authors:** J. T. Toguri, L. Grant-Nunn, R. Urquhart

**Affiliations:** 1 Dalhousie Medical School, Dalhousie University, Halifax, NS Canada; 2 Department of Emergency Medicine, University of Alberta, Edmonton, Alberta Canada; 3 Nova Scotia Health Authority, Halifax, Nova Scotia Canada; 4 Department of Community Health and Epidemiology, Dalhousie University, Room 8-032, Centennial Building, 1276 South Park Street, Halifax, Nova Scotia B3H 2Y9 Canada

**Keywords:** Cancer, Advance care planning, End of life, Qualitative

## Abstract

**Background:**

Advance care planning (ACP) is a process by which patients reflect upon their goals, values and beliefs to allow them to make decisions about their future medical treatment that align with their goals and values, improving patient-centered care. Despite this, ACP is underutilized and is reported as one of the most difficult processes of oncology. We sought to: 1) explore patients’ and families’ understanding, experience and reflections on ACP, as well as what they need from their physicians during the process; 2) explore physicians’ views of ACP, including their experiences with initiating ACP and views on ACP training.

**Methods:**

This was a qualitative descriptive study in Nova Scotia, Canada with oncologists, advanced cancer out-patients and their family members. Semi-structured interviews with advanced cancer out-patients and their family members (*n* = 4 patients, 4 family members) and oncologists (*n* = 10) were conducted; each participant was recruited separately. Data were analyzed using constant comparative analysis, which entailed coding, categorizing, and identifying themes recurrent across the datasets.

**Results:**

Themes were identified from the patient / family and oncologist groups, four and five respectively. Themes from patients / families included: 1) positive attitudes towards ACP; 2) healthcare professionals (HCPs) lack an understanding of patients’ and families’ informational needs during the ACP process; 3) limited access to services and supports; and 4) poor communication between HCPs. Themes from oncologists included: 1) initiation of ACP discussions; 2) navigating patient-family dynamics; 3) limited formal training in ACP; 4) ACP requires a team approach; and 5) lack of coordinated systems hinders ACP.

**Conclusions:**

Stakeholders believe ACP for advanced cancer patients is important. Patients and families desire earlier and more in-depth discussion of ACP, additional services and supports, and improved communication between their HCPs. In the absence of formal training or guidance, oncologists have used clinical acumen to initiate ACP and a collaborative healthcare team approach.

## Background

Advance care planning (ACP) is a process in which a patient reflects upon their goals, values and beliefs, allowing them to make decisions about their future medical treatments that align with their goals and values [1]. ACP is multifaceted, addressing the patient’s legal rights, who their substitute decision maker(s) will be, and their preferences for future medical procedures/interventions; thus it is beneficial for healthcare professionals (HCPs) to be involved in this process [2]. Due to its complicated nature and the involvement of multiple stakeholders, including patients, family members and medical staff, a systematic approach should be implemented during ACP discussions to ensure these conversations are initiated and that patients have a thorough understanding of their future care options [3]. ACP is associated with increased documentation of patients’ end-of-life (EOL) wishes, and improved quality of EOL care and patient/family satisfaction [3, 4]. Additionally, family members of patients who have undergone ACP report decreased stress, anxiety and depression during the bereavement period [3]. Despite these positive outcomes, less than 50% of elderly Canadian patients have engaged in ACP [5]. A Canadian study from 2014 to 2015, across three provinces, found that only 23% of individuals older than 50 had engaged in multiple aspects of ACP [6]. Similar findings have been reported elsewhere. For example, in the United States, from 2000 to 2012 there were no changes in the proportion of individuals who had engaged in EOL discussions, while there was an increase in patients who had defined a durable power of attorney [7]. This highlights there has been little increase in the number of patients who have engaged in ACP over time, despite research demonstrating the beneficial outcomes of ACP for both patients and family members as highlighted above [3, 8].

Given their poor prognosis and anticipated trajectory of decline, advanced cancer patients are ideal candidates for ACP [9–11]. Despite this, studies have shown low numbers of this patient population have had such discussions with their healthcare teams [12, 13]. The National Roundtable Proceedings on Advance Care Planning in 2009 argued it was “imperative” to discuss ACP when a patient had < 12 months to live [14]. Prior to this, an Ontario study demonstrated that less than 7% of their study population, patients with metastatic cancer in a palliative radiotherapy program, had a documented CPR code status in the patient chart [12]. These numbers are similar to a study conducted in the United States, in 2010, that documented as few as 9% of advanced cancer patients had discussed ACP with their oncologist [13]. Interestingly 48% of patients reported their desire to have this discussion with their oncologist, while 35% preferred to engage in ACP with their family physician [13]. Although studies have been conducted about ACP within the advanced cancer population, these have largely been quantitative investigations with limited qualitative studies [11]. Yet, such research is important to understand how we can best initiate, frame, and support these conversations in specific patient populations. This study addresses views on ACP in advance cancer populations from both patient, family, and oncologist perspectives, providing a comprehensive and holistic view on the subject.

For at least three decades, it has been reported that both patients and families believe in and recognize the importance of ACP, specifically individuals who are in poor health and dying but do not feel comfortable raising this issue with the healthcare team [15]. Additional barriers to engaging in ACP include stigma around EOL [16] and personal factors such as a patients attitudes, confusion, fear [17] and cultural and religious differences [18]. From a HCPs perspective, only 5% report feeling adequately prepared to explain advanced life sustaining treatments to patients [19], while qualitative studies report inadequate training [16] and misconceptions that ACP may alter patients’ hope and increase their anxiety [20], despite evidence in the literature to suggest otherwise [21].

This study investigated patient, family, and physician views on ACP within the context of advanced cancer patients. The objectives were to: 1) explore patients’ and families’ understanding, experience and reflections on ACP, as well as what they need from their physicians during the process; and 2) explore physicians’ views of ACP, including their experiences with initiating ACP and views on ACP training.

## Methods

### Research design & data collection

This was a descriptive qualitative study using in-depth, semi-structured, one-time one-on-one interviews [22]. Interviews were conducted either in person or via telephone, with patients who had advanced cancer, family members of patients with advanced cancer, and medical and radiation oncologists between May, 2016 and November, 2018.

Interviews were conducted by L.G.N or J.T., using an interview guide (Supplemental File 1). These guides were developed by the researchers, based on the research objectives and relevant literature, and adapted for participant group but sought perspectives on the following topics: participants’ understandings of ACP, perspectives on ACP, initiation of ACP, and improving ACP discussions. The interviewers had no prior relationship with any of the participants. All interviews were audiotaped and transcribed verbatim by an experienced transcriptionist. Even if participants were able to define ACP, all were given the same definition so they could reflect upon their experience equally. The definition given was “ACP is a process by which a patient, alongside his/her healthcare team, considers options about future healthcare decisions. The process includes clarifying a patients’ understanding of his/her treatment options and identifying his/her values and wishes for future medical care.” Neither L.G.N. or J. T had previous experience in qualitative interviewing, however, they were mentored by R.U. who has extensive experience in qualitative interviewing and qualitative research. At the time of data collection, J.T. and L.G.N. were medical students with limited experience in ACP. R.U. is a health services researcher with a program of research in ACP and goals of care discussion for persons with serious and life-limiting illness. Ethical approval to conduct the study was obtained from the Nova Scotia Health Authority Research Ethics Board. The Consolidated Criteria for Reporting Qualitative Research were followed [23].

### Participants

Participants were patients who had advanced cancer, family members of patients with advanced cancer, and medical and radiation oncologists in Nova Scotia, Canada. To be eligible for inclusion, patients (i) had advanced cancer (at diagnosis or recurrence) or (ii) had cancer and self-identified as a suitable candidate for ACP due to their health/disease circumstances (e.g., multi-morbidity, frailty). Family members had to have a close family member (e.g., spouse, sibling, child) with advanced cancer. Patients and family members self-identified as having advanced cancer; a particular cancer stage and/or diagnosis was not sought. All participants were recruited separately; none were dyads. These individuals were recruited by an experienced research coordinator and L.G.N. Recruitment involved posting study information and posters in the Nova Scotia Cancer Centre at the Queen Elizabeth II Hospital, Halifax, Nova Scotia and the Cape Breton Cancer Centre, Sydney, Nova Scotia and distributing to relevant organizations such as the Cancer Patient Family Network, senior’s councils/groups, community health boards, and local cancer charities*.* Posters were entitled, “Do you have advanced cancer? Would you like to talk about your health care planning needs and preferences?” and “Does someone in your family have advanced cancer? Would you like to talk about health care planning needs and preference for your family member?” Further details about the study, including that we were seeking participants views, needs, and preferences for future health care planning were included on the poster. Interested persons contacted the research coordinator or L.G.N. to express their interest and obtain additional information.

Staff medical and radiation oncologists from the Department of Medical Oncology and the Department of Radiation Oncology at the QEII Hospital in Halifax and Cape Breton Regional Hospital in Sydney were initially approached by R.U. or J.T. via email with an introduction to the study and invitation to participate. The same researcher followed up 1 week later for those who failed to respond. Of those contacted, 67% (10/15) were interviewed, all staff interviewed were from the QEII Hospital in Halifax, Nova Scotia. Staff were not specifically linked to patient or family member participants. All oncologists held active positions at a hospital and participated in patient care.

### Analysis

Thematic analysis was used to analyze the data, which entailed coding, categorizing, and identifying themes recurrent across the datasets [24]. Patient/family member interviews were analyzed as one dataset, while oncologist interviews were analyzed as a separate dataset. The goal of each analysis was to identify patterned responses or meanings in each dataset. The analytic process involved reading and rereading transcripts, applying a coding scheme to the transcribed text, and grouping the coded text into overarching abstract categories and themes. This was an inductive, iterative process, which entailed asking questions about what is and is not understood in the dataset and recoding the data when necessary. Two authors independently analysed the data and organized it into themes [RU, JT]. Data were discussed between authors and a consensus was reached on coding, themes, and thematic saturation. Data collection continued until no new themes emerged from the datasets.

## Results

Four patients, four family members, and ten oncologists participated in this study. One patient participated from the province of Ontario, where she was receiving treatment. This research was approved by the Nova Scotia Health Authority Research Ethics Board. None of the patient-family member participants were dyads. The duration of each interview was 38–64 min for the patient and family interviews, while oncologist interviews lasted from 26 to 69 min. Analysis of the transcribed interviews resulted in four overarching themes from patients/family members and five overarching themes from oncologists. Themes identified from patients/family members were: 1) positive attitudes towards ACP; 2) HCPs lack an understanding of patients’ and families’ informational needs during the ACP process; 3) limited access to services and support within the healthcare setting; and 4) poor communication between HCPs. Themes identified from oncologists were: 1) initiating ACP discussions; 2) navigating patient-family dynamics; 3) limited formal training in ACP; 4) ACP is better suited to / requires a team approach; and 5) lack of coordinated systems hinders ACP. These themes are described below; Tables 1 and 2 present illustrative quotations.

**Table 1:** Patient and Family Views

Overarching Theme	Illustrative Quote
Positive attitudes towards ACP	Advance care planning is what the patient desires for their last phase on this earth and how they wish to leave this world. [Participant 1] It [ACP] should be brought up hopefully long before a critical time …. And if one (patient or physician) doesn’t bring it up, the other one (patient or physician) should, even for a young person. Like if you go in for a physical … To me, that’s a time to discuss it and certainly with any, not even necessarily critical, but potentially serious medical problem. [Participant 2]
HCPs lack an understanding of patients’ and families’ informational needs during the ACP process	There was no plan. He barely got in there (the hospital) and then everything just fell apart. So, it would have been nice maybe if we’d known more about this (the cancer/the patient’s prognosis), maybe he could have had something happen at home and that would have been fine. Or maybe what did happen could have happened for him a little bit earlier so we could have been a little bit more at peace. … And even when they were explaining all this stuff to us, it actually was taking away time that we spent with my husband because the whole family was away spending like an hour or even an hour and a half finding out about it … that really wasn’t the time to find out about. [Participant 3] ACP means to me; do I want to be resuscitated if my heart stops? And I had asked for and signed a DNR order. [Participant 2]
Limited access to services and supports	...it is nice to have the support but sometimes it is almost more stressful having people [family members] there who are getting as upset as you or more. Suddenly then you have to be worried about their feelings. [Participant 1]. And they [ACP conversations] did help me to know what would happen in the future and how she would progress, and what some of the signs and signals would be from her [the patient]. Like bodily functions and just things like that for me to be aware of so I wouldn’t be afraid. [Participant 6] They [the hospital] have support groups and support workers available. I did have some trouble accessing them at the beginning of my treatment. That is what I kind of had the most need for. And I tried a couple of times to get a hold of them and never heard back and left a message that was forward or something like that. I think that there could be closer relationships between the medical team and the psychosocial oncology team. [Participant 1]
Poor communication between HCPs	Even though it was kind of implied and assured that it was a collaborative approach, to be honest, it seemed more like a step by step – Okay, I’ve done my part, I’m passing the baton. [Participant 3] I truly believe that the physicians need to get … they need to get on track with one another. They need to be communicating between themselves. Because they’re not doing that. One physician might know something, a specialist knows something else. But not being a team where they’re communicating together and they’re planning together. A patient should not have to go to a specialist and take her information with her to the specialist because the physician, the family physician didn’t inform them, or vice versa. [Participant 6]

**Table 2:** Oncologists Views

Overarching Theme	Illustrative Quote
Initiation of ACP discussions	… the majority of my patients, 99% of my patients are presenting with an incurable disease. But they’re treated. I don’t discuss those issues until I feel that there are no more treatment options, or the patients cannot tolerate the treatment and we should stop. Then comes the conversation about advance planning. [Participant 7]
Navigating patient-family dynamics	So that’s the downside of advance planning. It’s actually different for the patient and the family. So, it’s actually often much more accepted by the patient. And the patient comes to terms with it a lot quicker than the family. And there’s many … You know, one of the difficulties is the patient who comes in here and their family wants to be aggressive, and they don’t. [Participant 3]
Limited formal training in ACP	… so we have a case … We’ve inserted some videos in there. … we’ve introduced the concept. The same method I learned about breaking bad news. So it’s in there. It’s the only mandatory reading actually. And then some videos on people doing it well. [Participant 10] So that’s part of the teaching. I let them carry on the conversation and telling me what they think and how would they approach. But that’s their learning pattern. This is very informal, okay. But it’s with the patient …. At some point they will develop their own style, yes. [Participant 7]
ACP requires a team approach	… somebody shows up and really nothing has been done, and then I look in the system and I’m like, you know what, this person has seen like 3 surgeons, has seen the rad onc [sic], has seen all these … What were they all doing? You know, why should it be left to me to fix everything in one appointment? Well, I cannot fix everything in one appointment. So I prioritize and I try and do what I can. But if I can’t get everything done in one appointment, especially in someone who I know has been seeing lots of other people, well, they’re going to come back to see me. [Participant 8] What’s very interesting is they [patients] can be 90 years old and they’ll have never talked about it [ACP]. Which again, it doesn’t matter if you’ve got cancer or not, the fact that no family physicians ever brought it up with them either is interesting. [Participant 10] It’s always proven that the early involvement of a supportive care team or a palliative care team has such a huge impact in terms of patient satisfaction but also in terms of management issues in terms of quality of life and even in terms of survival. [Participant 6]
Lack of coordinated systems hinders ACP	It would be helpful to have one provincial approach that’s appropriately communicated to all healthcare providers so that we can consistently in the same way communicate to patients and family members …. There should be one source of documentation, right … That we have that advance care planning documentation on there so that with one click, we know. Right now we have to go to 5 or 6 different health records, and then we still may not get it.” [Participant 6]

### Patient and family views

#### Positive attitudes towards ACP

Almost all patients and family members portrayed positive attitudes towards ACP. Although most participants were unable to specifically define ACP, this appeared due to unfamiliarity with the term itself. When given the definition, they expressed that they had indeed participated in activities and discussions that are part of ACP. All participants understood ACP involved planning around the end-of-life care. Patients and families felt ACP discussions increased their knowledge about the disease process, allowing an opportunity to plan for future medical emergencies, as well as the practical aspects of living with a serious illness (e.g., finances) toward which they expressed positive feelings. ACP was felt to empower patients, allowing them to regain a sense of control during a time when much of their autonomy had been stripped from them. Personal experiences with ACP ranged from individuals with metastatic cancer who had undergone conversations around their wishes for pain/symptom management as they continued non-curative intent treatment to family members who expressed negative experiences near their end of life due to the lack of timely conversations and an advance care plan. Most participants stated ACP should occur shortly after their diagnosis of cancer, with one individual expressing that ACP should be discussed prior to any serious diagnosis.

In the context of advanced cancer, participants expressed their beliefs that is it the responsibility of the physician to initiate these discussions. They also expressed their views that HCPs must provide patients and families the time and space to assimilate feelings and emotions about prognosis and not feel rushed to make decisions.

#### HCPs lack an understanding of patients’ and families’ informational needs during the ACP process

Patients reported feeling they were not fully involved in decision-making processes during their care. This occurred as patients and families thought their HCPs did not know or appreciate what information they required to make informed decisions. Specifically, participants described wanting all the information possible to evaluate options and inform their own care plans. They also emphasized wanting to know their prognosis at an earlier stage and what to expect. Some participants reported physicians not discussing all aspects of their care and many perceived their ACP and goals of care (GOC) discussions were conducted too late in their disease trajectory. Specific aspects of care patients deemed particularly important were earlier access to palliative care and the possibility of transitioning care to home. Some reported a desire for additional discussions on topics such as the changes in medications as they neared EOL. A lack of these conversations deeply and negatively affected patients and families, resulting in distrust and frustration with the healthcare system. One participant described a situation whereby a failure to discuss prognosis and what to expect earlier in the disease trajectory resulted in limiting time with a loved one prior to death.

Participants also discussed how the transfer of information was sometimes impeded by medical jargon. This jargon either had no meaning to the patient or their family, or the term had permeated into the lay population without being properly defined, such as the terms palliative care and ACP. Participants said a common misconception was that palliative care referred only to hospice and EOL care and had no association with symptom management. Most participants had never heard of ACP prior to the study. Those who believed ACP was synonymous with a do-not-resuscitate order did not appreciate that ACP involved discussion of one’s values and preferences for care, encompassing all aspects of treatment at the EOL. Limitations in communication were felt to disrupt the quality of health care that patients and their families received.

#### Limited access to services and supports

Participants felt they had limited access to the services and supports they needed for ongoing discussions concerning prognosis and future care, including conversations about the extent to which healthcare professionals and healthcare services could be involved. Approximately half of the participants discussed limited access to the psychosocial supports they needed to engage in and support comprehensive ACP. While participants mentioned receiving emotional and psychological support from family, friends, and their communities of faith, they expressed concern with exclusively using their personal support networks as they believed that doing so resulted in additional stress. Both patients and family members discussed these social supports as important to decreasing stress around discussing their values and preferences. At the same time, patients highlighted that formal supports would have been helpful for both themselves and their family members prior to the initial ACP discussion to emotionally prepare them for the conversations. In fact, several patient participants stated their preferences that family members not take part in ACP discussions because family members might react more negatively to these discussions than the patient, require additional supports, and cause the patient additional distress that he/she/they (the patient) would prefer to avoid. Conversely, family participants stated that involvement in ACP conversations relieved stress as they then knew how to appropriately plan for the patients’ inevitable decline in health.

During discussions with physicians, some participants, primarily patients, were referred to formal supports in the healthcare system as part of the ACP process, including counselors, spiritual care and palliative care. However, many participants felt the needed individuals and services were difficult to access through the cancer centers/clinics.

Family members reflected on the challenges aligning care with the patient’s preferences, particularly with respect to the use of home care services and palliative treatment at home. Participants believed these topics were not discussed in a timely manner that would have benefited the patient.

#### Poor communication between HCPs

Participants discussed their perception that there was poor communication between members of their healthcare teams. This was described in terms of ongoing breakdowns in communication between referring physicians, medical oncologists, radiation oncologists, the palliative care team, and professional psychosocial supports. Patients felt their care was transferred from one specialist / subspecialist with limited handover of patient information (or values). Others described negative experiences where they did not feel that there was any communication between specialists.

### Oncologist views

#### Initiation of ACP discussions

Many participants defined their ACP discussions with patients as any conversation involving the topic of disease course, prognosis, treatment (non-curative intent), EOL care, and GOC. These conversations often focused on discussing DNRs, symptom management and quality of life. Participants’ experiences initiating ACP were described as complex processes that encompassed a number of variables, including how family members could influence patients ACP decisions and the timing of the discussion. One of the salient variables was whether patients and families had already thought about ACP. For those patients who had not previously heard of ACP or had not considered what their advance care plan may include, oncologists said they decided on when to initiate the ACP discussion based on their clinical acumen. This clinical acumen was also used to determine the extent of information they disclosed to patients. They discussed that this process is typically determined by extent of the disease, prognosis and how they believed the patient and family would tolerate the conversation.

#### Navigating patient-family dynamics

Participants discussed that ACP often involved navigating patient-family relationships, which either facilitated the process or significantly inhibited it. As in all aspects of healthcare, there is often not one “patient” and discussions about treatment, GOC, and EOL decisions were often complicated by family members involved in the process. Participants discussed the various dynamics between families that made ACP less or more challenging, including situations whereby patients and families had discussed plans prior to seeing the oncologist and engaging in ACP (facilitating the process) to situations where patients had accepted their illness while their family members had not (hindering the process). Pertaining to the latter situations, some oncologists discussed navigating these conversations by reminding those present that ACP was to determine the wishes of the patient and not others. Conversely, some stated they would not interject as they did not want to cause a disruption in the family or alter the family dynamic going forward. The decision of how oncologists would react to any family pressure were made on a case-by-case basis.

#### Limited formal training in ACP

Most participants had no formal training in delivering ACP. Participants indicated that medical education around ACP was (during their training) and remains (today) minimal. They described the formal education at the current undergraduate level as primarily involving learning how to “break bad news”, which is not ACP. Oncologists reflected on how they taught ACP to their residents, which was similar to how they were taught, through informal education in clinical settings. Processes were described as clinical learning where individuals witnessed others performing ACP, forming their own “style” that incorporated aspects that the individual deemed to be important or facilitate ACP and disregarding those they thought impeded ACP. Some participants discussed changes that could be instituted to increase training of ACP, including but not limited to simulation and increasing teaching around cancer and its prognosis.

#### ACP requires a team approach

Participants emphasized that a team approach is crucial in ACP and identified several other members of the healthcare team who are integral to discussing and developing a patient’s advance care plan: other physicians (surgeons and family doctors), allied healthcare (nurses, social work, nutritionists), spiritual care, the palliative care team (if involved), and the psychosocial oncology team. Oncologists viewed the team approach as crucial for a number of reasons, most prominently time constraints and competing clinical responsibilities*.* There was a unifying view that each member of the healthcare team was important but also that each must play a specific role in ACP as to limit misinformation or lack of accountability. This was highlighted by many participants who discussed that patients can be told multiple, and sometimes conflicting, information. Oncologists identified several activities as being part of their specific role, including sharing important clinical information such as prognosis, helping patients identify goals of care, particularly treatment goals, and referral to other HCPs as warranted (e.g., palliative care, social work, and psychosocial oncology).

Despite appreciating others involvement, some participants were critical about how other members of the healthcare team approached ACP and their specific role in the process. This was largely related to initial oncology consultations with patients who had been referred from another provider without having had a discussion related to prognosis, clinical / pathological results, or ACP.

Participants expressed mixed views on the role of primary care providers (PCP) in ACP. Some participants believed PCP should play an integral role in ACP due to the nature of the patient-physician relationship. Additionally, many commented that ACP would be an appropriate conversation for PCP to be having with all older patients regardless of their health conditions. However, participants also expressed disappointment that such conversations had not occurred in primary care.

Participants expressed positive views on both the palliative care team and their early intervention. They described specifically referring patients to palliative care to further ACP discussions around practical (e.g., legal and financial) issues, even implying or explicitly stating that it would be beyond their scope of practice to have these discussions. Some participants believed it was solely the responsibility of palliative care to discuss EOL care and ACP. Similarly, participants emphasized the role of psychosocial teams (counselors, psychologists, spiritual counselors) to attend to patients’ anxieties and emotional/spiritual well-being. These auxiliary supports were positively viewed as they provided a skillset and training that participants did not feel they could provide.

In contrast to the overall positive attitude participants held towards palliative care and the psychosocial team, the role of nursing was not seen as central in ACP discussions. As one participant articulated, “*their (nurses’) role is different but very complementary*”. The nursing role was often discussed as an informal role with nurses acting as a sounding board for the patient. That is, nurses were viewed as professionals who can help patients frame questions or seek whether patients had thought about their EOL but were never to discuss prognosis or formalizing advance directives.

#### Lack of coordinated systems hinders ACP

Participants discussed an overall lack of coordination when it came to the healthcare system and how it functions. This ranged broadly from isolated approaches specialists take during care provision to the need to improve documentation systems. The system itself was perceived to be largely reactive to patient problems versus proactive and preventative. The segmentation of services was described by Participant 8 as “… *each one of us putting up silos*.” Participants further described the fractioning of the healthcare system as leading to burnout and complacency. Participants described this lack of coordinated systems as hindering ACP discussions and leading to a fragmentation of services, which resulted in increased frustration amongst HCPs. Moreover, participants believed that improved documentation of a patient’s advance care plan would be one mechanism to enable direct lines of communication between the silos that exist between providers and institutions. They felt this would improve both informational, relational and management continuity for patients with advanced disease.

## Discussion

This study investigated the views and experiences of ACP from two stakeholder groups: advanced cancer patients and their families, and oncologists. It was identified that ACP was positively viewed by patients and families; however, there was a discrepancy between what patients wanted to know and the information they received prior to making informed ACP decisions. Despite the overall positive attitude, patients and families expressed concerns that there was a lack of accessibility to HCPs as well as a lack of communication between HCPs.

Oncologist expressed difficulties initiating ACP and using their clinical acumen to determine when they believed it was appropriate. They experienced ACP as difficult when navigating conflicting preferences between patients and their family. Oncologists identified a lack of training in ACP, from undergraduate medical education to continuing professional development. They also identified areas that could be actively targeted to improve ACP. One area of strength was the use of a team approach. Despite the advantages of a team approach, there were also notable challenges, including the need to improve system-level functioning. This study provides important insight into ACP processes and experiences across multiple stakeholder groups. Despite an awareness of the importance of ACP since at least the mid 1990s [25] and the more recent movement to a person-centred care model, there has been limited research conducted on what advanced cancer patients think about ACP or their views on how ACP is performed. Instead, studies have investigated the views and practices of clinicians on ACP [26].

### Commonalities and differences in views

Similar themes arose in both datasets. All stakeholders perceived ACP to improve patient care. All discussed existing limitations to providing optimal ACP due to lack of services and supports, whether that was access to HCPs and needed services or a lack of coordination across providers and systems. Additionally, both groups identified communication between HCPs as suboptimal. Patients and families referred to a lack of relational continuity, or the continuity of the therapeutic relation between the patient and healthcare provider(s), as well as decreased informational continuity [27] as concerns impacting ACP, while oncologists expressed problems with disrupted informational and management continuity. Whereas patients and families desired earlier initiation of ACP, increased knowledge about their disease process, and more through discussion of future care options, oncologists believed these discussions occur when appropriate following their evaluation of a multitude of factors. Systems-wide, oncologists discussed several challenges and stated their desire for increased accessibility to and standardized documentation of advance care plans.

### Comparison with existing literature

The positive attitudes towards ACP demonstrated in this study has been found by others for older patients and those with advanced illness, including but not limited to cancer [28–31]. Nonetheless, some research has reported that patients and families do not want to discuss ACP due to fear of death or not wanting to break their optimism [32], or because patients did not fully understand their illness and/or prognosis [28]. Regarding the notion of breaking optimism, advanced cancer patients provided with the opportunity to engage in ACP had no significant changes in hope, hopelessness, or anxiety [21]. While most research exploring patient views on ACP come from North America and Europe, interestingly, Taiwanese patients expressed views similar to patients in this study, despite cultural differences and norms. Patients expressed their beliefs that ACP consults were helpful to understand the course of their disease, rights around treatment, and treatment choices, and reported relief that they had a future plan [28].

HCPs typically acknowledge and accept the benefits of ACP in terms of improving person-centred care [33]. However, EOL discussions have been described as one of the most difficult aspects of oncology [34, 35]. In this study, oncologists reported feeling comfortable initiating ACP discussions and discussing treatment during EOL but, at the same time, reported difficulty with extending these conversations to include the non-medical aspects of ACP (e.g., legal issues). When they do initiate these conversations, participants described doing so based on their own clinical acumen. Similar experiences have been described in other studies [36, 37]. Kim et al. showed that the physician’s interpretation of a patient prognosis was a key factor in initiating ACP discussions in primary care [36]. Despite this, a Canadian study of ACP and EOL plans in elderly individuals, who were at a high risk of death in 6 months, described that although > 75% of patients had thought of EOL care, only 47% had completed ACP and less than 30% had discussed their wishes with their family physician. Approximately 55% had discussed their wishes with a HCP [5]. Arguably, ACP discussions may be occurring late in the disease course if they are left until a palliative care referral. Indeed, patients and family members in this study suggested that when ACP does occur, it is often too late to put plans in place to ensure a patient’s preferences are met. A recent Nova Scotia-based study on palliative care consultation and aggressive EOL decisions in unresectable pancreatic cancer demonstrated that > 25% of patients received a “late” (more than 8 weeks following diagnosis) palliative care consult, and > 15% received no palliative are consult. All patients lived less than 1 year [38].

Given the recognition that many patients do not engage in these discussions with their healthcare teams, tools and resources have been designed and tested to support patients and HCPs in having these discussions. The Serious Illness Conversation Program from Ariadne Labs is one tool for clinicians that has been used throughout the United Sates and has begun to permeate Canadian healthcare, such as in British Columbia [39, 40]. Similarly, Huang et al., tested a three-step structured process to support physicians in engaging in ACP with their advanced cancer patients: 1) an initial discussion about treatment choices and options; 2) a consult with palliative care specialist (if the patient desired) giving possible benefits and negatives of treatment choices alongside resources to help patients explore the EOL preferences; and 3) deliberation about the options available and documentation [41]. Documentation of DNR changed from 0% following the initial talk, to 44.3% after the second part, to 80.9% following the final discussion. Following the decision talk, 80% of the patients felt their EOL goals had been discussed [41]. Definitive protocols and tools/resources that support ACP appear particularly useful in terms of ensuring these conversations occur rather than relying on clinical acumen to do so. Future research should investigate the best strategies to implement such protocols and resources in practice, so they are routinely used in the course of clinical care.

Still, the provision of protocols and resources to support ACP may not lead to physicians initiating these conversations if they lack the confidence or skillset to do so. As oncologists in this study discussed, they do not necessarily have the skillsets to manage the psychosocial aspects of advanced cancer care, nor do they necessarily feel ACP falls within their scope of practice. Moreover, oncologists discussed referring patients to palliative care and psychosocial oncology teams for conversations around the practical and emotional aspects of ACP, suggesting they (or at least some of them) are uncomfortable having such holistic discussions. These views may reflect a lack of confidence and training in ACP. Fulmer et al., found that physicians experience many barriers to ACP, including disagreements between family members (65%), feeling uncomfortable with the conversation (51%), not knowing the right timing (60%) and a lack of training in ACP [26]. Less than 29% of physicians surveyed in that study reported any formal ACP training. There is limited literature discussing physician ACP training. However, those who have tested training sessions have observed improved patient outcomes, including ACP documentation [28] and decreased patient anxiety and depression following ACP training with the Serious Illness Care Program for clinicians working with oncology patients [42, 43].

Oncologists in this study discussed expectations around other HCPs, particularly PCPs whom they felt should play a larger role in ACP. Oncologists felt that PCPs should be having ACP conversations with all older patients, regardless of health status, yet they do not have these conversations with all their own patients who have been diagnosed with a life-limiting condition and instead describe relying on their own clinical acumen. It is possible that oncologists feel PCPs are better equipped, having long-standing relationships with many patients, to initiate discussions around a person’s values and preferences for future medical care than oncologists, and rather see themselves as being in a better position to discuss prognosis and treatment goals. Studies investigating ACP in primary care demonstrate an area for improvement. A Canadian study found PCPs were willing and confident in engaging in ACP discussions, but were not often doing so [44]. The same study investigated non-physician HCP willingness, confidence, and engagement in ACP. They reported high confidence and willingness amongst non-physicians to engage in ACP discussions, yet a situation whereby these professionals almost never initiated these conversations. A review on PCP and nurse involvement in EOL describes an active role in ACP, yet noted they were less likely to be involved if the patient had cancer [45]. Within oncology, palliative care nurses have been shown to increase the number of ACP discussions as well as hospice referrals [46]. Oncologists in this study perceived the role of nurses in ACP as limited. However, some jurisdictions such as British Columbia, Canada, are training nurses to engage in ACP, with modifications to delivering prognosis [39, 40]. With appropriate training, non-physician HCPs may play an important role in these conversations and alleviate some of the pressures placed on physicians to initiate ACP.

### Limitations

This study has several limitations. As it was conducted in Nova Scotia, Canada, it may not be generalizable to other jurisdictions. However, the purpose of qualitative research is not to be generalizable but to bring forward insights that improve our understanding of particular phenomena. Additionally, the small sample size, particularly for patients/families, and that they self-identified for participation, may mean that we did not capture the full range of patient/family perspectives and experiences. However, our findings align with the literature in this area, suggesting the findings are indeed transferable. Another limitation is that most participating patients/family members had limited experience with ACP, though most mentioned taking part in activities or discussions that constitute some part of ACP. This means that the emotions and experiences these conversations can provoke may not have been fully captured in this study because participants had limited concrete experiences on which to draw. Nevertheless, participants did have advanced cancer or cared for someone with one, and therefore also had their personal experiences with the diagnosis and prognosis to reflect on, including their expectations of and interactions with their health care team*.* Finally, we did not capture the views of other oncology providers (nurses, allied health, social work) who may in fact initiate ACP with advanced cancer patients and have differing views and experiences than oncologists on this topic. Further research should investigate the experiences of these HCPs as well as the role they could play in ACP.

## Conclusion

This study highlights important views from different stakeholders on ACP for the advanced cancer patient. We identified that patients and family members appreciate ACP, however they believe these conversations should occur earlier in the disease course and involve a more thorough discussion of treatments, options and EOL care, allowing them to feel their informational needs have been met. Patients discussed a lack of services and supports, which they perceived as limiting ACP and the ability to meet their EOL-care goals. An additional obstacle to optimal ACP was a lack of communication between HCPs, resulting in decreased informational continuity. Similar to patients and family members, oncologists appreciated the importance of ACP. They reported the need for a healthcare team approach to ACP. Oncologists identified areas of weakness with respect to initiating ACP, such as their ability to navigate the patient-family dynamic. They recognized overarching challenges to provide patient-centered ACP, including limited formal training in ACP and a lack of coordinated systems.

## Supplementary information

**Additional file 1.** Interview guide for patients and families of advanced cancer.

## Data Availability

The datasets used and analyzed during the current study are available from the corresponding author on reasonable request.

## Abbreviations

ACP: Advanced care planning
DNR: Do not resuscitate
EOL: End-of-life
GOC: Goals of care
HCPs: Healthcare professionals
PCP: Primary care providers
QOL: Quality of life
