# Supplementary material for: Views of advanced cancer patients, families, and oncologists on initiating and engaging in advance care planning: a qualitative study
Source: BMC Palliat Care. 2020 Oct 1;19:150. doi: 10.1186/s12904-020-00655-5 (PMC7531150; doi:10.1186/s12904-020-00655-5)
Supplement: Supplementary file 1 — Additional file 1. Interview guide for patients and families of advanced cancer. [file 12904_2020_655_MOESM1_ESM.docx]

**Interview guide for patients and families of advanced cancer**

*Beginning / setting the stage*

1. Tell me about your illness.
   1. What do you understand about your cancer?
   2. Are you receiving treatment right now? If so, what type?
2. Tell me about what your needs are at this point. [Probes: disease management, physical (pain, other symptoms, functioning), psychological (depression, anxiety, distress, fears), social (relationships with family/friends, privacy, isolation), spiritual, practical (personal care, dependents, transportation), legal, end of life care/planning]
3. Have you talked to a doctor or other healthcare provider, such as a nurse, who is specialized in palliative care? If so, how did you feel about those discussions?

*Advanced care planning*

1. Tell me about your understanding of advanced care planning.
   1. Have you ever heard of this term? [*If no, interviewer to describe; if yes, interviewer to clarify/expand on ACP if/when warranted*]

ACP is a formal decision-making process in which a patient reflects upon their goals, values and beliefs to allow them to make better decisions about their future medical treatment. ACP often includes discussing end-of-life care including if a patient would like a do not resuscitate (DNR) order, the extent of medical treatment to prolong life, the type of pain management, assigning substitute decision maker(s) and many other important questions

1. What are your thoughts on this type of planning?/
   1. Potential benefits? Potential concerns?
2. Thinking back to some of the things we discussed earlier – for example, [*specify needs identified in Q3*] – how do you think advanced care planning could address some of those needs? [Probes: understanding what to expect, comfort of knowing a plan is in place if necessary, completing things, reducing anxiety, relieving family burden]
3. Has anyone from your care team ever brought up these types of discussions with you? If so, who? What did he/she say? How did you and your family feel during these discussions?

*Physicians’ actions and behaviors*

1. How would you like your doctor or health care team to bring up these discussions?
   1. At what time do you feel these discussions are appropriate?
   2. Who would you prefer start these discussions – for example, your family doctor, your cancer specialist, your cancer nurse or social worker?
   3. Who else would you want to be involved? [Probes: spouse, child, spiritual or religious advisor]
2. How could your doctor best support you and your family during these discussions? [Probes: provide information (e.g., about symptom and pain control, home supports, daily activities, psychological health, spiritual needs, legal issues); communicate clearly and honestly; ask about/discuss fears and worries; provide reassurance and confirmation; be available between visits/discussions to answer questions; anticipate needs and plan for them (e.g., place of care, dependent/caregiver care, respite care, emergency care)]
3. What about when making decisions about your wishes for future medical care – for example, when making decisions about your goals of care, about what medical treatments you want to receive or not (e.g., life support), or about who you would want to make your healthcare decisions if, for some reason, you cannot?
   1. What would you need from your doctor to help you make these kinds of decisions?
4. Can you describe to me the ‘best-case scenario’ in terms of how your doctor can support you through these decisions?

*Closing*

**Interview guide for oncologists**

*Demographic questions*

1. How long have you been working with patients with advanced cancer?
   1. Has this always been in Nova Scotia?

*Advance care planning*

1. Tell me about your understanding of advance care planning.
   1. Have you ever heard of this term? [*If no, interviewer to describe; if yes, interviewer to clarify/expand on ACP if/when warranted*]
2. What are your thoughts on this type of planning?
3. Potential benefits? Potential concerns?
4. What role do you typically play in advance care planning? [Probes: disease management, physical (pain, other symptoms, functioning), psychological (depression, anxiety, distress, fears), social (relationships with family/friends, privacy, isolation), spiritual, practical (personal care, dependents, transportation), legal, end of life care/planning]
5. If no, what roles do you think you should be involved in?
6. Are there additional roles you can think of?
7. How long do you typically spend talking to a patient about advance care planning?
8. How comfortable do you feel talking to patients about advance care planning?
9. What do you talk to patients and families about when discussing advance care planning?
10. What situation/do you do this in [formal setting, one on one, with the family, at the bedside]
11. Do you feel patients/families respond to discussions about advance care planning?
12. Tell me about a time you feel the advance care planning process went particularly well.
    1. What was it about that situation that made it successful?
13. Have you ever had formal education with respect to advance care planning? Where was this training [Probes: was it during school or post-graduation, where did you go to school, how long ago was this training, if not what informal training have you received on advance care planning]

*Improving Advance Care Planning*

1. Do you think that you have the appropriate resources in which to learn more about advance care planning [courses, electives, emotional training, informal mentor who can teach you about advance care?]
2. Do these conversations about advance care planning occur with other healthcare professionals or are their team goals which you strive for?
3. Do you feel like the rest of the healthcare team is appropriately trained for advance care planning?
4. How do you think advance care planning could be improved upon?

*Closing*

1. Is there anything at all we have not talked about that you believe is important to discuss with respect to advance care planning? If so, what?
